# Dangguijakyak-San Protects against 1-Methyl-4-phenyl-1,2,3,6,-tetrahydropyridine-Induced Neuronal Damage via Anti-Inflammatory Action

**DOI:** 10.1155/2013/976270

**Published:** 2013-08-31

**Authors:** Deok-Sang Hwang, Hyo Geun Kim, Jun-Bock Jang, Myung Sook Oh

**Affiliations:** ^1^Department of Oriental Gynecology, College of Korean Medicine, Kyung Hee University, No. 1 Hoegi-dong, Dongdaemun-gu, Seoul 130-701, Republic of Korea; ^2^Department of Oriental Pharmaceutical Science and Kyung Hee East-West Pharmaceutical Research Institute, College of Pharmacy, Kyung Hee University, No. 1 Hoegi-dong, Dongdaemun-gu, Seoul 130-701, Republic of Korea

## Abstract

Dangguijakyak-san (DJS), a famous traditional Korean multiherbal medicine, has been used to treat gynecological and neuro-associated disease. Recent studies demonstrated that DJS has multiple bioactivities including neuroprotection. In the present study, we were to investigate the effect of DJS and its mechanism in an *in vitro* and *in vivo* model of Parkinson's disease (PD). In primary mesencephalic culture system, DJS attenuated the dopaminergic cell damage induced by 1-methyl-4-phenylpyridine toxicity, and it inhibited production of inflammatory factors such as tumor necrosis factor **α** (TNF-**α**), nitric oxide (NO), and activation of microglial cells. Then, we confirmed the effect of DJS in a mouse PD model induced by 1-methyl-4-phenyl-1,2,3,6-tetrahydropyridine (MPTP). In the pole test, DJS at 50 mg/kg/day for 5 days showed increase of motor activity showing shortened time to turn and locomotor activity compared with the MPTP only treated mice. In addition, DJS significantly protected nigrostriatal dopaminergic neuron from MPTP stress. Moreover, DJS showed inhibition of gliosis in the substantia nigra pars compacta. These results have therapeutic implications for DJS in the treatment of PD via anti-inflammatory effects.

## 1. Introduction

 One of most common neurodegenerative diseases is Parkinson's disease (PD), which is characterized pathologically by the selective, irreversible loss of dopaminergic (DA) neurons in the substantia nigra pars compacta (SNpc) and their terminals in the striatum and clinically by bradykinesia, resting tremor, rigidity, and disturbances in posture and gait [1].

The initial factors that cause neuronal death remain unclear. Studies have suggested that the pathology of PD involves oxidative stress [2, 3], apoptotic mechanisms [4], mitochondrial dysfunction [5], and the accumulation of toxic protein [6, 7]. Other studies have shown that dopaminergic neuronal death is related to inflammatory processes involving increases in inflammatory mediators, including tumor necrosis factor-*α* (TNF-*α*), interleukin-1*β*, and interferon-*γ* [8, 9], and the activation of glial cells [10]. Recent studies have suggested that anti-inflammatory therapy might be an effective therapeutic strategy for neuroprotection [11].

Dangguijakyak-san (DJS), also called Danggui-Shaoyao-San (DSS) or Toki-shakuyaku-san (TJ-23), is a widely used traditional Korean herbal medicine consisting of Paeoniae Radix, Cnidium Rhizome, Alismatis Rhizoma, Angelicae Gigantis Radix, Poria, and Atractylodis Rhizoma Alba. In biological studies, DJS has hormone-like [12], antianemia [13], and antihypertensive [14] effects. Also, DJS stimulated progesterone production in rat luteal cells and luteal steroidogenesis [15], improved the quality of life of postmenopausal women in Korea [16], and improved motor activity after peripheral facial nerve axotomy [17]. Additionally, studies have revealed that DJS has neuro-associated actions; DJS attenuated cognition problems and had regulatory effects on the central cholinergic nervous system in scopolamine-treated mice [18], DJS protected cortical neurons against amyloid *β*-induced neurotoxicity [19], and DJS improved cognitive function and protected ultrastructure of brain cortex in aged mice [20]. Moreover, DJS protected dopaminergic cells from hydrogen peroxide and 6-hydroxydopamine-induced neurotoxicity *in vitro* and against 1-methyl-4-phenyl-1,2,3,6,-tetrahydropyridine- (MPTP-) induced damage in estrogen-deprived mice by inhibiting oxidative stress such as reactive oxygen species production and glutathione level depletion and suppressing apoptosis through mitochondria-mediated caspase pathway [21–23]. 

Based on those actions of DJS, we hypothesized that DJS might be a potent neuroprotective agent in PD model. However, the effect of DJS on neuroinflammation, one of therapeutic target for PD, has not been investigated yet in animal models. Therefore, we examined the protective effect of DJS against 1-methyl-4-phenylpyridinium- (MPP+-) induced neurotoxicity and explored its possible mechanisms by measuring inflammatory factors in rat primary dopaminergic cells. Then, we confirmed the protective effect of DJS in a mouse model of PD by performing behavioral tests and a histological analysis.

## 2. Materials and Methods

### 2.1. Materials

Minimal essential medium (MEM) and fetal bovine serum (FBS) were purchased from Gibco Industries (Auckland, New Zealand). Glucose, l-glutamine, paraformaldehyde (PFA), MPTP, MPP+, poly-l-lysine (PLL), 3,3-diaminobenzidine (DAB), Griess reagent, sodium chloride, sucrose, and phosphate-buffered saline (PBS) were purchased from Sigma-Aldrich (St. Louis, MO, USA). The rat tumor necrosis factor-alpha (TNF-*α*) ELISA kit was purchased from Invitrogen (Carlsbad, CA, USA). Affinity-purified rabbit anti-tyrosine hydroxylase (TH) polyclonal antibody, mouse and rat anti macrophage-1 antigen integrin alpha M (MAC-1) affinity-purified monoclonal antibody, and rabbit anti glial fibrillary acidic protein (GFAP) affinity-purified monoclonal antibody were obtained from Merck Millipore (Billerica, MA, USA). Biotinylated anti-rabbit, anti-mouse, and anti-rat antibodies, normal goat serum, and a standard avidin-biotin peroxidase complex (ABC) kit were purchased from Vector Laboratories (Burlingame, CA, USA). The DJS extract was the same as that used in previous studies [22, 23].

### 2.2. Primary Culture of Mesencephalic Dopaminergic Cells

Cultures were prepared from the mesencephalons of gestational day 14 embryos from Sprague-Dawley rats, obtained from Dae Han Biolink (Eumseong, Korea). The ventral mesencephalon was separated, dissected, pooled, dissociated, and plated on 24-well plates with coverslips precoated with PLL at a density of 1.5 × 10^5^ cells per well. Cultures were maintained in a humidified incubator in an atmosphere of 5% CO_2_ in air at 37°C in MEM with 6.0 g/L glucose, 2 mM glutamine, and 10% FBS. On the 6th day *in vitro* (DIV 6), the medium was changed to serum-free MEM, and the cells were treated with DJS (0.04 or 0.2 *μ*g/mL) for 1 h and then stressed with MPP+ (15 *μ*M) for 23 h. Then, the cells were fixed with 4% PFA at room temperature for 30 min. The cells were stored in PBS at 4°C for immunocytochemistry.

### 2.3. NO Assay

The accumulation of nitrite in the culture supernatant was determined with a colorimetric assay using Griess reagent (0.1% N-(1-naphthyl) ethylenediamine dihydrochloride, 1% sulfanilamide, and 2.5% H_3_PO_4_). Equal volumes of culture supernatant and Griess reagent were mixed and incubated for 10 min at room temperature in the dark. The absorbance at 540 nm was determined spectrophotometrically (SpectraMax Gemini EM; Molecular Devices, Sunnyvale, CA, USA). The NO concentration was determined from the sodium nitrite standard curve. Control cells were treated in the same way without DJS and MPP+, and the absorbances were expressed as percentages of the control.

### 2.4. TNF-*α* Assay

The TNF-*α* assay was performed in accordance with the manufacturer's instructions. Briefly, 50 *μ*L of culture supernatant was mixed with 50 *μ*L of incubation buffer. To all samples, 50 *μ*L of standard diluent buffer was added. These samples were pipetted with 50 *μ*L of biotin conjugate, and the side of the plate was tapped gently to mix it. The plates were incubated for 60 min with a plate cover at room temperature. After aspirating the liquid, all wells were washed four times. Then, 100 *μ*L of streptavidin-horseradish peroxide working solution was added and incubated for 45 min with a plate cover at room temperature. After aspiration and washing, 100 *μ*L of stabilized chromogen was added to the wells. The plates were allowed to stand for 30 min at room temperature in the dark. Next, 100 *μ*L of stop solution was added, and the side of the plate was tapped gently to mix it. The plates were read using a spectrophotometer at 450 nm. The standard smooth curve was plotted with fitting software using a four-parameter algorithm, and the data are expressed as standard rat TNF-*α* (pg/*μ*L).

### 2.5. Animals

Animal maintenance and treatment were carried out in accordance with the Principles of Laboratory Animal Care (NIH publication number 85-23, revised 1985) and the Animal Care and Use Guidelines of Kyung Hee University, Seoul, Korea. Eight-week-old male C57BL/6 mice were purchased from Dae Han Biolink. The animals were housed at an ambient temperature of 23 ± 1°C and relative humidity 60 ± 10% under a 12 h light/dark cycle and were allowed free access to water and food.

### 2.6. Drug Administration

Animals were assigned to three groups: (1) control (*n* = 9; intraperitoneal and intraoral vehicle); (2) MPTP (*n* = 9; intraperitoneal MPTP plus intraoral vehicle); and (3) MPTP + DJS (*n* = 9; intraperitoneal MPTP plus intraoral DJS) groups. DJS dissolved in saline was administered at 50 mg/kg/day for 5 days. MPTP (base form) in normal saline was injected at 20 mg × 4/kg/day at 2 h intervals for the last day of DJS treatment.

### 2.7. Behavioral Test and Brain Tissue Preparation

To measure motor coordination, we performed the pole test the day after the last MPTP injection. The mouse was placed head upward near the top of a vertical rough-surfaced pole (diameter 8 mm, height 55 cm). The times it took for the mouse to turn completely downward (time to turn; T-turn) and then to reach the floor (locomotion activity time; T-LA) were recorded, with a cut-off limit of 30 s. Then, mice were sacrificed on the first and seventh days to quantify inflammatory and dopaminergic factors, respectively. The mice were anesthetized with 50 mg/kg Zoletil (intramuscularly) and rapidly perfused transcardially with PBS, followed by 4% PFA in 0.1 M phosphate buffer (PB). Then, the brains were removed rapidly, postfixed in 4% PFA solution, and processed for cryoprotection in 30% sucrose at 4°C. The frozen brains were cut into 30 *μ*m coronal sections using a cryostat microtome (CM3000; Leica, Wetzlar, Germany). Finally, the tissues were stored in storing solution containing glycerin, ethylene glycol, and PB at 4°C for immunohistochemistry.

### 2.8. Immunocytochemistry and Immunohistochemistry

Primary cells on cover slips and free-floating sections were rinsed in PBS at room temperature before immunostaining. They were pretreated with 1% hydrogen peroxide in PBS for 15 min to remove endogenous peroxidase activity. Then, they were incubated overnight at room temperature with a rabbit anti-TH antibody (1 : 2,000 dilution) to detect dopaminergic neurons, a mouse or rat anti-MAC-1 antibody (1 : 1000 dilution) to detect microglial cells, and a rabbit anti-GFAP antibody (1 : 1000 dilution) to detect astrocytes. Next, they were incubated with a biotinylated antisecondary IgG for 90 min and then in ABC solution for 1 h at room temperature. The peroxidase activity was visualized with DAB for 4 min. After every incubation step, the cells and tissues were washed three times with PBS. Finally, the primary cells on cover slips were mounted on gelatin-coated glass slides, air dried, and photographed through a microscope (Axioskop 2; Carl Zeiss, Göttingen, Germany). The free-floating brain tissues were mounted on gelatin-coated slides, dehydrated, cleared with xylene, and cover slipped using Histomount medium. To quantify the effect of DJS on the mesencephalic dopaminergic and microglial cells, TH- and MAC-1-immunopositive cells were counted on at least four cover slips from independent experiments for each condition. The effect of DJS on brain tissues was quantified by counting the number of TH-immunopositive cells in the SNpc at ×100 magnification under a microscope. The TH-immunoreactivity in the striatum (ST) was measured at ×40 magnification using a Stereo Investigator (MBF Bioscience, Williston, ND, USA). The anti-inflammatory effects of DJS were visualized with MAC-1 and GFAP-immunopositivity in the SNpc at ×400 magnification under a microscope. Data are presented as percentages of control group values.

### 2.9. Statistical Analysis

The data are expressed as means ± standard errors of the mean (SEM). Data were analyzed by one-way analysis of variance (ANOVA) followed by the least significant differences (LSD) using SPSS 12.0 K for Windows (SPSS, Chicago, USA). For all statistical analyses, *P* < 0.05 was considered significant.

## 3. Results 

### 3.1. DJS Protects Dopaminergic Cells against MPP+-Induced Toxicity in Rat Primary Mesencephalic Culture System

**Figure 1:**
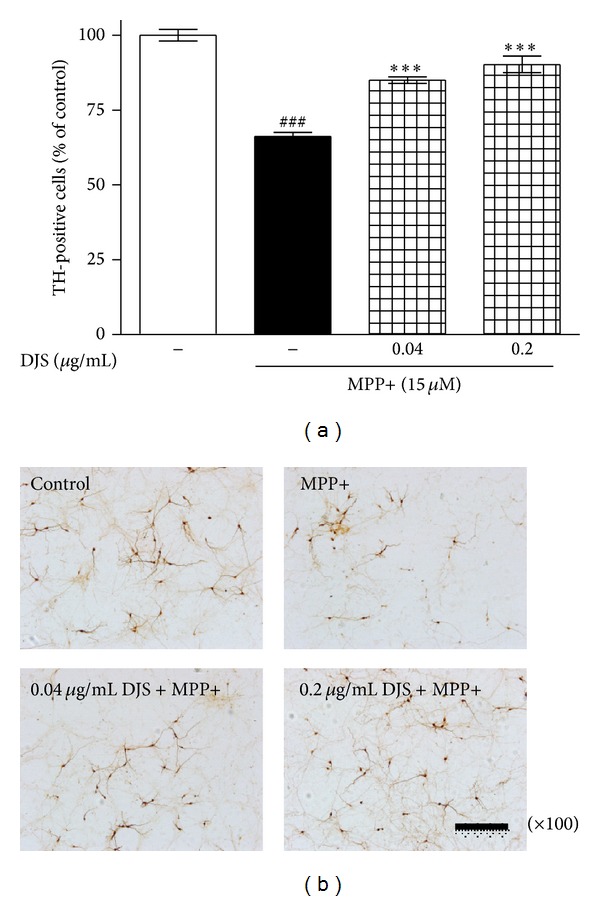
Protective effect of DJS against MPP+ neurotoxicity in primary mesencephalic cells. After cells were treated with DJS and 15 *μ*M MPP+, dopaminergic neurons were stained with a TH antibody. The number of TH-positive cells was counted (a) and the representative images were shown (b). Scale bar = 100 *μ*m. Each column represents the mean ± SEM from four replications. Data are expressed as percentages relative to the controls. ^###^ *P* < 0.001 significantly different from the control group. ***P < 0.001 significantly different from the MPP+ only treated group.

To investigate the effect of DJS on neuroprotection against MPP+ toxicity in mesencephalic neuronal cells, we counted dopaminergic cells. MPP+ neurotoxicity was defined as a 66.20% reduction in the survival rate compared with the control group. Treatment with 0.04 and 0.2 *μ*g/m DJS L increased the survival of dopamine cells to 85.05 and 90.25%, respectively, compared with the control group (Figure 1).

### 3.2. DJS Inhibits Gliosis Induced by MPP+ in Rat Primary Mesencephalic Culture System

**Figure 2:**
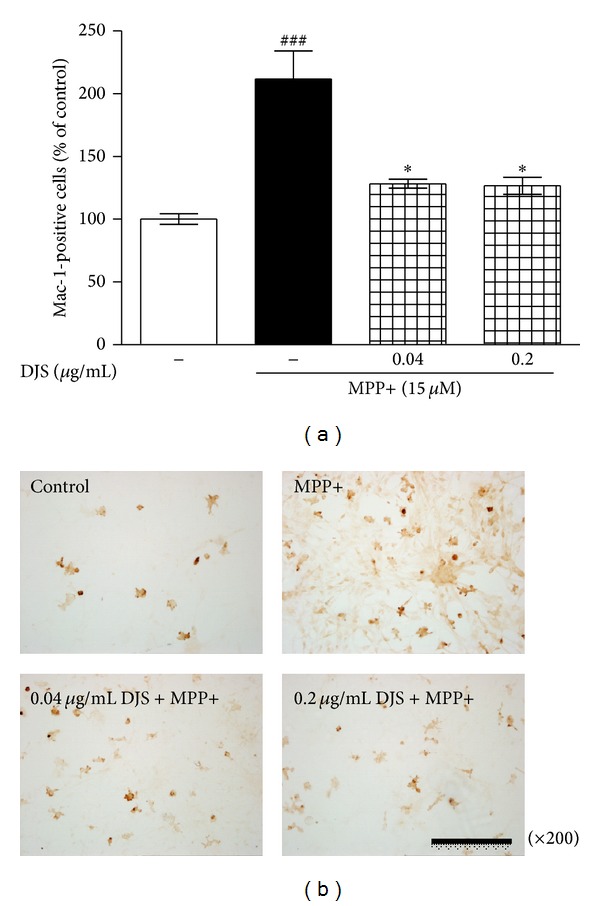
Inhibitory effect of DJS on MPP+-induced gliosis in primary mesencephalic cells. After cells were treated with DJS and 15 *μ*M MPP+, microglia cells were stained with a Mac-1 antibody. The number of Mac-1-positive cells was counted (a), and the representative images were shown (b). Scale bar = 200 *μ*m. Each column represents the mean ± SEM from four replications. Data are expressed as percentages relative to the controls. ^###^ *P* < 0.001 significantly different from the control group. *P < 0.05 significantly different from the MPP+ only treated group.

To determine the protective effect of DJS on MPP+-induced microglia activation, we counted MAC-1-positive cells. Treatment with 15 *μ*M MPP+ increased active microglia cells by 211.76% compared with the control group, whereas treatment with 0.04 and 0.2 *μ*g/mL DJS significantly inhibited it to 128.29 and 126.64%, respectively, compared with the controls (Figure 2).

### 3.3. DJS Inhibits Neuroinflammation Induced by MPP+ in Rat Primary Mesencephalic Culture System

**Figure 3:**
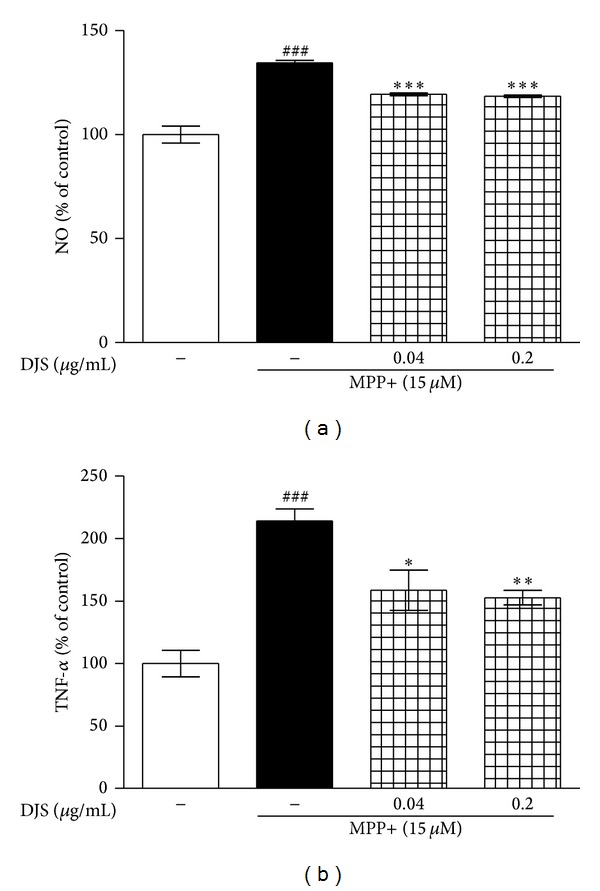
Inhibitory effects of DJS on MPP+-induced NO and TNF-*α* productions in primary mesencephalic cells. After cells were treated with DJS and 15 *μ*M MPP+, the supernatants were activated with Griess reagent and rat TNF-*α* ELISA kit. The nitrite contents (a) and TNF-*α* level (b) were determined. Each column represents the mean ± SEM from four replications. Data are expressed as percentages relative to the controls. ^###^ *P* < 0.001 significantly different from the control group. ***P < 0.001, **P < 0.01, and *P < 0.05 significantly different from the MPP+ only treated group.

To assess the protective effect of DJS on MPP+-induced neuroinflammation involving NO and TNF-*α* production, Griess reagent and a rat TNF-*α* ELISA kit were used, respectively. The incubation of cells with 15 *μ*M MPP+ increased NO production by 134.48% compared with the control group, whereas treatment with 0.04 and 0.2 *μ*g/mL DJS significantly decreased it to 119.37 and 118.44%, respectively, compared with the control (Figure 3(a)). Additionally, treatment with MPP+ increased the TNF-*α* level by 214.13% compared with the control, whereas the 0.04 and 0.2 *μ*g/mL DJS treatments inhibited TNF-*α* production by 158.57 and 152.63%, respectively (Figure 3(b)).

### 3.4. DJS Protects Movement Impairment Induced by MPTP in a Mouse Model of PD

**Figure 4:**
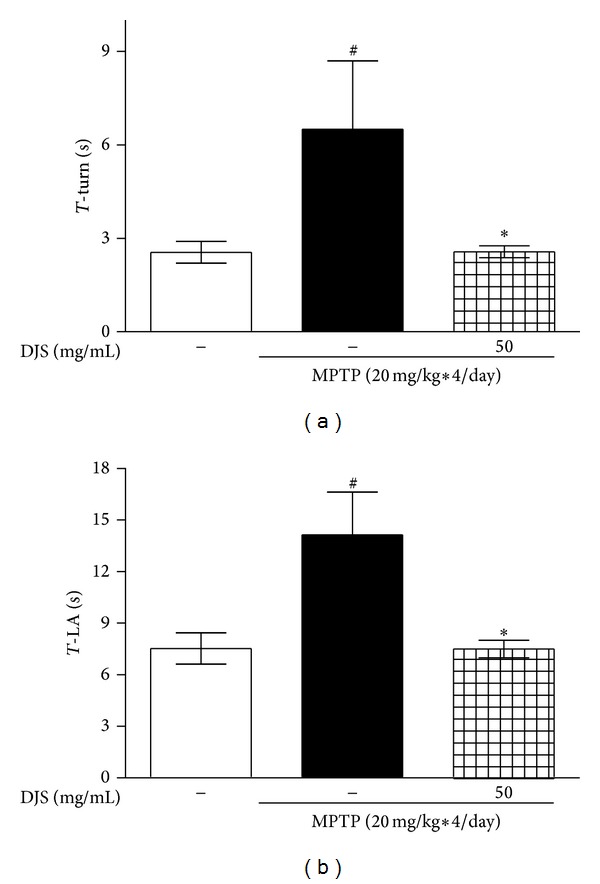
Inhibitory effect of DJS on MPTP-induced bradykinesia in a mouse model of PD. DJS at 50 mg/kg/day for 5 days was administered, and MPTP at 20 mg/kg was injected four times at 2 h interval on the day of last DJS treatment. On the first day of MPTP injection, the pole test was performed. The times to turn (T-LA) and the times to completely downward and to arrive at the floor (b) were recorded. Each column represents the mean ± SEM from ten replications. Data are expressed as percentages relative to the controls. ^###^ *P* < 0.001 significantly different from the control group. ***P < 0.001, *P < 0.05 significantly different from the MPTP only treated group.

To examine whether DJS could relieve the motor symptoms in the MPTP-induced mouse PD model, we performed the pole test. In this test, the MPTP-only treated group showed bradykinesia, with the time to turn at the top (T-turn) and time to climb down (T-LA) prolonged by 254.90 and 187.90%, respectively, compared with the control. However, DJS treatment at 50 mg/kg/day for 5 days reduced this to 100.78 and 99.60%, respectively, compared to the control (Figure 4).

### 3.5. DJS Protects Dopaminergic Neuronal Damage and Gliosis Induced by MPTP in a Mouse Model of PD

**Figure 5:**
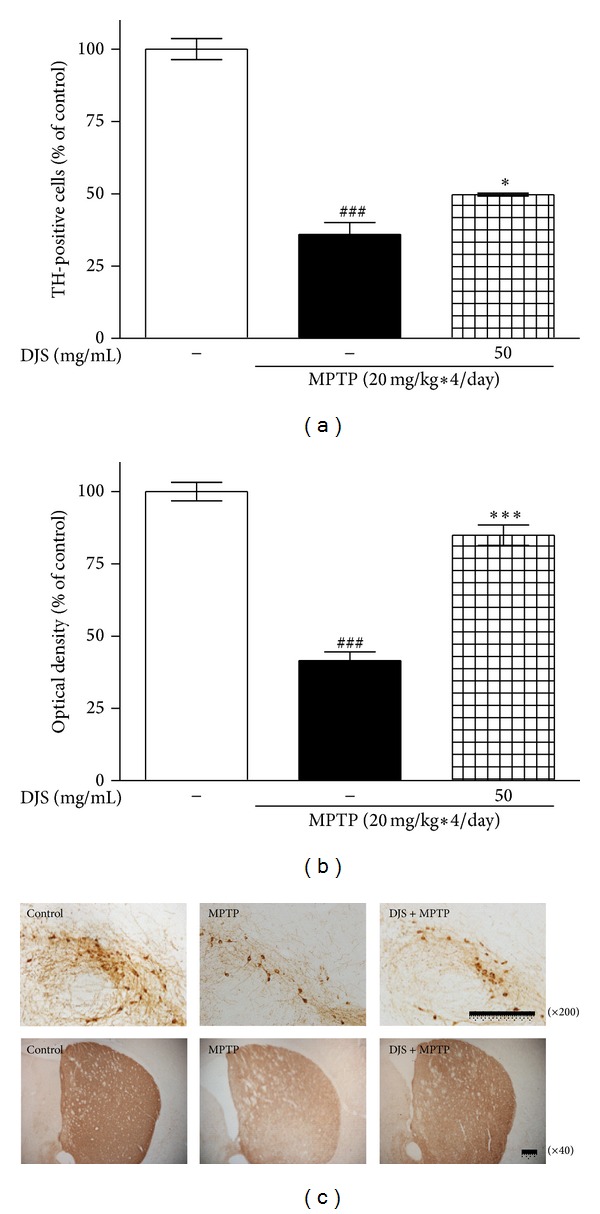
Protective effect of DJS on MPTP-induced dopaminergic neuronal damage in a mouse model of PD. DJS at 50 mg/kg/day for 5 days was administered, and MPTP at 20 mg/kg was injected four times at 2 h interval on the day of last DJS treatment. On the seventh day of MPTP injection, dopaminergic neurons and fibers in the SNpc and the ST, respectively, were stained with a TH antibody. The number of TH-positive cell bodies was counted (a), and the optical density of TH-positive fibers was measured (b). The representative images were shown (c). Scale bar = 100 *μ*m. Each column represents the mean ± SEM from six replications. Data are expressed as percentages relative to the controls. ^###^ *P* < 0.001 significantly different from the control group. ***P < 0.001, *P < 0.05 significantly different from the MPTP only treated group.

**Figure 6:**
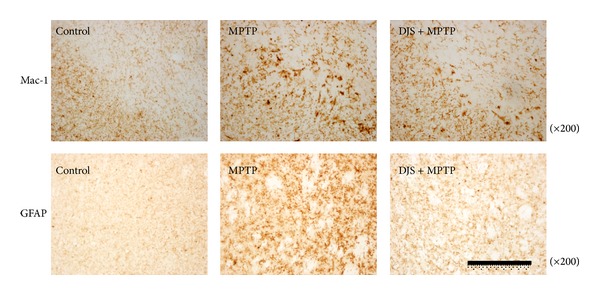
Inhibitory effect of DJS on MPTP-induced gliosis in a mouse model of PD. DJS at 50 mg/kg/day for 5 days was administered, and MPTP at 20 mg/kg was injected four times at 2 h interval on the day of last DJS treatment. On the first day of MPTP injection, microglia and astrocyte in the SNpc were stained with Mac-1 and GFAP antibodies, respectively, and the photographs were shown.

The protective effects of DJS on dopaminergic neurons in a mouse PD model induced by MPTP were also investigated. Seven days after MPTP treatment, the number of TH-positive cell bodies in the SNpc was decreased by 35.91%, and the optical density of the TH-positive fibers in the ST was decreased by 41.45%. The administration of DJS at 50 mg/kg/day for 5 days protected the nigrostriatal dopaminergic neurons, reducing loss by 49.69 and 84.89% in the SNpc and ST, respectively, compared with the control (Figure 5). Additionally, we found that MAC-1- and GFAP-immunopositive cells were increased in the MPTP only treated group, whereas they were decreased in the DJS treatment group (Figure 6). These results show that DJS reversed the toxic effect of MPTP on neuronal cells by inhibiting inflammatory factors.

## 4. Discussion

This study evaluated the neuroprotective effects of DJS against MMP+/MPTP toxicity and the mechanism of the effect. The treatment of DJS led to significant neuroprotection of dopaminergic neurons against MMP+/MPTP-induced stress and inhibited microglia activation and inflammatory factor production *in vitro* and *in vivo*. To understand the mechanism of therapy, it is important to study microglial activation in the pathogenesis of PD. It has been reported that activated microglia cells actively participate in the pathogenesis of MPTP-induced PD via the release of cytotoxic factors [24]. We observed that exposure to MPP+ resulted in a significant increase in the proinflammatory factors MAC-1, NO, and TNF-*α* in the cultures and altered cell morphology. The elevation of MAC-1, which is expressed exclusively on microglia in the central nervous system, has been reported in the MPTP model of PD [25]. Furthermore, MAC-1 contributes to the MPP+/MPTP-induced reactive microgliosis and progressive dopaminergic neurodegeneration, so inhibiting microglial cells might constitute a novel microglia-suppressive therapy for the treatment of PD [26]. In this study, DJS inhibited MAC-1 against MPP+-induced gliosis. We performed a TNF-*α* assay to investigate whether DJS exerts anti-inflammatory activity on mesencephalic dopaminergic cells. TNF-*α* might damage dopaminergic cells directly by activating an intracellular death pathway, leading to the production of toxic amounts of NO [11]. The NO levels were determined to assess the effects of DJS on NO generation. Mesencephalic dopaminergic cells exposed to MPP+ generated increased NO, whereas DJS treatment significantly reduced this enhancement. The pathogenesis of PD involves impaired mitochondrial function, inflammation, and oxidative damage [27]. Free radicals such as reactive oxidative species (ROS) and NO can cause oxidative damage, which can attenuate microglia proliferation [28]. Furthermore, excessive NO production in the brain contributes to the neuronal energy deficiency in PD [29]. In the primary mesencephalic culture treated with MPP+, DJS attenuated the loss of TH-positive neurons and decreased the generation of MAC-1-positive cells, TNF-*α*, and NO.

Furthermore, we performed the pole test to determine the effect of DJS on the behavior symptoms of PD. We used C57BL/6 mice, which are susceptible to MPTP toxicity and are useful for studying the protective and compensatory mechanisms of PD [30]. In the pole test, T-turn and T-LA were prolonged significantly in the MPTP-treated mice, whereas they were shortened significantly in the mice treated with DJS before MPTP. The pole test is a measure of bradykinesia and is very sensitive to nigrostriatal dysfunction [31, 32]. We also demonstrated that DJS can protect against behavior problems in ovariectomized mice [23]. It was reported that herbs containing Paeoniae Radix improved the movement disorder [33]. As DJS contains Paeoniae Radix, DJS likely inhibited the toxic effect of MPTP, that is, bradykinesia. DJS improved behavior, which is in agreement with the findings using the Morris water maze test and step-down passive avoidance test [34].

In mice, the anti-inflammatory actions of DJS resulted in a significant decrease in dopaminergic cell death induced by MPTP. Activated microglia and reactive astrocytes to a lesser extent are found in the area associated with cell loss, possibly contributing to the inflammatory process by the release of proinflammatory prostaglandins or cytokines [31]. In the MPTP-treated mice, severe shrinkage of the cell bodies and a decrease in dopaminergic cells and optical density were observed in the SNpc and ST. By contrast, the dopaminergic cells in the DJS mice remained intact. Additionally, MPTP increased striatal astrocytes and microglial cells in the SNpc, whereas DJS treatment attenuated the two markers of activated microglia. These results show that DJS reversed the toxic effect of MPTP on neuronal cells in the SNpc.

Our study showed that DJS has a neuroprotective effect on dopaminergic cells via anti-inflammatory effects. Recently, it was reported that DJS has an antidiabetic effect via its antioxidative properties [35]. Previously, we showed that DJS protects against the glutathione decrease induced by 6-OHDA and inhibits ROS production [22]. We also reported that DJS has neuroprotective effects via antioxidant and antimitochondria-mediated apoptotic effects [22, 23]. In this study, we showed that the neuroprotective effect of DJS could be attributed to the inhibition of MPTP-stimulated microglial activation by inhibiting TNF-*α* and NO. Therefore, DJS has anti-inflammatory effects as well as antioxidant and antiapoptotic effects.

These anti-inflammatory effects of DJS may be related to the effects of each constituent herb. Angelicae Gigantis Radix has been reported to have anti-inflammatory activity and to inhibit the production of pro-inflammatory cytokines, including TNF-*α*, IL-6, and IL-8 [29]. Four compounds isolated from *Cnidium officinale* inhibited COX-2 and iNOS expressions in LPS-stimulated macrophages [27]. Paeoniflorin, a main component of Paeoniae Radix, has been reported to inactivate the inflammatory response by inhibiting activation of the NF-*κ* B pathway via the inhibition of I*κ*B kinase activity [36]. The interactions of these effects might result in the anti-inflammatory effect of DJS. PD has been associated with many factors, including environmental toxins, genetic factors, mitochondrial dysfunction, and oxidative stress. Furthermore, neuroinflammation is recognized as a major factor in the pathogenesis and treatment of PD [37]. DJS is a potential candidate for treating PD via multiple mechanisms.

In conclusion, DJS significantly improved the movement disorder in MPTP-induced mice and protected dopaminergic neurons. Additionally, an anti-inflammatory effect of DJS was seen as the mechanism by which DJS treatment attenuated the loss of dopaminergic cells. We believe that the anti-inflammatory effect of DJS may be useful for treating PD patients.
